# Bacterial Diversity in Pet Rabbits: Implications for Public Health, Zoonotic Risks, and Antimicrobial Resistance

**DOI:** 10.3390/microorganisms13030653

**Published:** 2025-03-13

**Authors:** Smaranda Crăciun, Cristiana Ştefania Novac, Nicodim Iosif Fiţ, Cosmina Maria Bouari, Lucia Victoria Bel, George Cosmin Nadăş

**Affiliations:** 1 Department of Microbiology, Immunology and Epidemiology, Faculty of Veterinary Medicine, University of Agricultural Sciences and Veterinary Medicine, 400372 Cluj-Napoca, Romania; smaranda.craciun@usamvcluj.ro (S.C.); nfit@usamvcluj.ro (N.I.F.); cosmina.bouari@usamvcluj.ro (C.M.B.); gnadas@usamvcluj.ro (G.C.N.); 2 New Companion Animals Veterinary Clinic, Faculty of Veterinary Medicine, University of Agricultural Sciences and Veterinary Medicine, Calea Mănăştur 3-5, 400372 Cluj-Napoca, Romania; lucia.bel@usamvcluj.ro

**Keywords:** rabbit, zoonotic, disease surveillance, Romania, multidrug resistance, one health

## Abstract

This study examined epidemiological aspects of rabbit pathologies, identified bacterial strains, and assessed their antimicrobial resistance, emphasizing rabbits as potential reservoirs for zoonotic multidrug resistant (MDR) bacteria and the need for continuous monitoring and antimicrobial stewardship. Samples from rabbits were cultivated and then identified using Vitek^®^ 2 and MALDI-TOF. Antimicrobial susceptibility was assessed by disk diffusion testing. This study analyzed 170 individuals with various pathologies, with males (58.24%) outnumbering females (41.76%). Dental abscesses (35.29%) and respiratory infections (28.24%) were most common. Antibiotic exposure was noted in 47.06% of cases, primarily involving trimethoprim (35.56%). Of the total samples, 91.18% tested positive, revealing 200 isolates from 23 bacterial genera, with *Staphylococcus* spp. (31%) and *Escherichia coli* (12%) being most frequently identified as well as species with zoonotic potential, such as *Pseudomonas aeruginosa*, *Staphylococcus aureus*, *Klebsiella pneumoniae*, *Proteus mirabilis*, and *Enterococcus faecium*. Antimicrobial susceptibility testing showed high efficacy for florfenicol (75%), ciprofloxacin (74.12%), and amikacin (68.65%), while significant resistance was found for kanamycin, neomycin, and trimethoprim. Nearly 49% of strains were MDR, with Gram-positive cocci, Enterobacteriaceae, and non-Enterobacteriaceae showing varying resistance, across 18 MDR genera. In conclusion, pet rabbits are potential reservoirs of zoonotic and MDR bacterial species, posing a risk for their owners.

## 1. Introduction

The domestic rabbit (*Oryctolagus cuniculus*) serves diverse functions, encompassing experimental research, meat production, exhibition showcasing, and, predominantly, companionship in numerous regions [1]. It holds the distinction of being the second-most common specialty pet among households in Europe and the USA [2]. The recent rise in pet rabbit ownership in Europe and North America highlights the importance of understanding the diseases they carry, as this knowledge is vital for treating sick rabbits and protecting public health from potential zoonotic transmission [3].

Bacterial diseases are common in pet rabbits, affecting various body systems. Successful treatment involves accurate diagnosis, addressing husbandry issues, and managing concurrent diseases [4]. Dental disorders are one of the most frequent causes of veterinary consultation for these pets, followed by gastrointestinal, dermatological, ocular, and respiratory pathologies [5].

Common dental issues in rabbits, such as the overgrowth of cheek teeth and incisors, lead to malocclusions, abscesses, dacryocystitis, and stomatitis, often causing significant morbidity and mortality [6,7,8]. The bacteria involved in the physiopathology of dental abscesses are associated with both anaerobic and aerobic bacteria, including *Fusobacterium*, *Peptostreptococcus*, *Bacteroides*, *Pseudomonas aeruginosa*, *Pasteurella* spp., *Streptococcus* spp., *Staphylococcus* spp., *Actinomyces* spp., *Proteus vulgaris*, and *Escherichia coli* [9,10,11].

Rabbits are highly prone to respiratory illnesses, often caused by bacterial infections, with *Pasteurella multocida* being the primary pathogen. *Bordetella bronchiseptica* and *Moraxella catarrhalis* also contribute, with weanlings being more susceptible to *Bordetella*, while *Pasteurella* dominates in adults [12,13]. *Streptococcus agalactiae* causes severe respiratory distress in farm rabbits, while *Streptococcus intermedius* is linked to odontogenic abscesses and sinusitis in pets [14]. Other pathogens, isolated from cases of upper respiratory tract disease include *Pseudomonas* spp., *Staphylococcus* spp., *Moraxella catarrhalis*, *Escherichia coli*, *Proteus mirabilis,* and *Mycoplasma* spp. [15].

Dacryocystitis is common in pet rabbits, though ocular diseases are poorly documented [16]. *Pasteurella* species are the main pathogens in nasolacrimal ducts, with *Staphylococcus aureus*, *Corynebacterium* spp., *Pseudomonas aeruginosa*, *Moraxella catarrhalis*, *Staphylococcus epidermidis*, and *Escherichia coli* also frequently isolated. Blepharitis may involve *Treponema cuniculi* (rabbit syphilis), while *Pasteurella multocida* often causes dacryoadenitis, linked to immunosuppression and multifocal infections [17,18,19,20].

External otitis can cause purulent discharge and middle ear infections, often due to secondary bacterial infections. Bacteria may ascend through the Eustachian tube following upper respiratory diseases. Middle ear infections with *Pasteurella multocida* are common, but bacteria such as *Pseudomonas*, *Bordetella*, *Escherichia coli*, *Proteus mirabilis*, *Moraxella*, and *Mycoplasma* species may also be involved [15,21]. Other bacteria may include *Staphylococcus aureus*, *Corynebacterium lactis*, *Corynebacterium mastitidis*, and *Aeromonas salmonicida* [22,23].

Bacterial enteritis, a significant cause of mortality in adult rabbits, primarily affects neonates and juveniles under 16 weeks, especially under stress from weaning, transportation, or overcrowding [24]. Among the microbial causes of intestinal diseases, enterohemorrhagic *Escherichia coli* (EHEC), a zoonotic pathogen producing Shiga toxins, can cause hemorrhagic colitis and diarrhea [25]. Common syndromes include enterotoxemia, caused by *Clostridium spiroforme*, dysentery with multifactorial origins (*Escherichia coli*, *Clostridium* spp., coccidia), and Tyzzer’s disease, linked to *Clostridium piliforme* [26]. Other bacterial pathogens that cause enteritis in rabbits include *Salmonella* spp., *Klebsiella pneumoniae*, *Klebsiella oxytoca*, and *Pseudomonas aeruginosa* [24].

Skin conditions are common in rabbits, either as primary issues or secondary to stress, pain, or chronic diseases [27,28]. Common skin pathologies include pododermatitis, dermatitis, abscesses, and alopecia, with pododermatitis being the most frequent. Abscesses may occur anywhere on the body, often due to otitis externa, trauma, or bites [27]. Perineal moist dermatitis results from prolonged exposure to urine or feces, usually involving *Staphylococcus aureus*, though *Pseudomonas aeruginosa* can be present. Other bacterial isolates include *Pasteurella* spp., *Fusobacterium* spp., and *Streptococcus* spp. [2,28,29].

Antimicrobials are to be applied only when secondary or primary bacterial infections are confirmed or likely, with therapy guided by susceptibility tests and a precise diagnosis [30]. One of the most crucial factors in antibiotic therapy for rabbits is the adverse reactions to specific medicines at dosages that are generally safe for other species. Some antibiotics, like clindamycin, ampicillin, amoxicillin, cephalosporins, and erythromycin, can cause serious gastrointestinal issues by disrupting the bacterial balance, sometimes leading to fatal enteritis. Enrofloxacin and trimethoprim combinations are generally considered safe for long-term oral use [24,31,32].

A specific concern arises from the widespread use of enrofloxacin, the most common fluoroquinolone on the market. Since fluoroquinolones are classified by the World Health Organization as ’Highest Priority Critically Important Antimicrobials’ for human health, veterinarians must use them judiciously. This practice aims not only to reduce the emergence of resistance in animal patients, but also to weaken the potential transmission of antimicrobial resistance to human populations [33].

Antimicrobial resistance (AMR) is a global health threat, driven by overuse in both human and veterinary medicine, with veterinary practice contributing significantly to resistant pathogens. The close contact between pets and their owners creates opportunities for the transmission of pathogenic bacteria, including MDR (multidrug resistant) strains, which pose a major threat to both public and veterinary health. The “One Health” approach, emphasizing collaboration across human, animal, and environmental health, is crucial to combat AMR, as resistance can transfer between humans, livestock, and pets [2,34,35,36,37,38,39].

Certain bacterial species in small mammals pose significant zoonotic risks to pet owners, underscoring the One Health approach. Contact with pets increases exposure to pathogens like *Pasteurella multocida*, *Staphylococcus aureus*, *Pseudomonas* spp., *Streptococcus viridans*, *Escherichia coli*, *Klebsiella* spp., and *Bordetella bronchiseptica*. This emphasizes the need for collaboration between veterinary and human health professionals to prevent cross-species pathogen transmission and protect both public and animal health [40,41,42].

This study primarily focused on investigating key epidemiological parameters associated with various rabbit pathologies, alongside the isolation and identification of diverse bacterial strains, as well as their antimicrobial susceptibility testing. Furthermore, this study aimed to emphasize the critical role of rabbit populations as potential reservoirs for zoonotic pathogens, highlighting the public health implications of their capacity to harbor MDR bacteria. By addressing these aspects, this research contributes valuable insights into both veterinary and human health concerns, emphasizing the need for ongoing monitoring and responsible antimicrobial stewardship.

## 2. Materials and Methods

### 2.1. Animals and Study Design

This study was conducted between November 2022 and December 2024, consisting of 170 pet rabbits that were referred to the Exotic Animal Clinic, Faculty of Veterinary Medicine from Cluj-Napoca, Romania. Each individual was subjected to a clinical examination performed by the specialized medical staff, and different samples were sent to the department of Microbiology of the same faculty for further bacterial analysis. The specimens were collected using a sterile cotton swab with Amies transport medium (DeltaLab, Barcelona, Spain) for the following pathologies: otitis, conjunctivitis, dermatitis, gastrointestinal (GI) disorders, respiratory tract infections, dental disease, urinary tract infections, and others. Each sample was accompanied with a dispatch note containing data about the owner, the animal (breed, age, and gender), the season, the type of sample, and whether the patient underwent antibiotic treatment prior to the bacteriological exam or not. Each specimen was appropriately labeled, sent, and processed in the same day.

### 2.2. Microbial Identification

Upon receipt, the samples were registered, followed by microbiological processing. Inoculations were performed for all the samples using a streaking method on common and special culture media for pathogen detection: Columbia agar with 10% sheep blood (BioMaxima S.A, Lublin, Poland), UriSelect medium (Bio-Rad Laboratories Inc., Hercules, CA, USA), and MacConkey agar (Merck, Darmstadt, Germany). Plates were aerobically incubated at 37 °C for 24–48 h; after incubation, the colonies’ cultural characteristics were evaluated followed by microscopic examination using the Gram staining technique. The preliminary classification of the bacterial strains was performed using potassium hydroxide 3%, the slide catalase test with 3% hydrogen peroxide for Gram-positive cocci, and the oxidase test (Rotitest^®^ Oxidase strips, Carl Roth, Karlsruhe, Germany) for Gram-negative rods.

When no bacterial growth was observed after 48 h, the sample was considered negative. Following the preliminary identification, 24 h cultures were used for further testing using the Vitek^®^ 2 Compact 15 system (Biomerieux, Craponne, France) and then confirmed with the MALDI Biotyper^®^ Sirius System (Bruker, Ettlingen, Germany). Briefly, Vitek^®^ consists of evaluating 64 biochemical characters using reagent cards (GP for Gram-positive cocci and GN for Gram-negative bacilli). The method was performed according to the manufacturer’s instructions. On the other hand, MALDI Biotyper^®^ uses mass spectrometry to identify bacterial strains by comparing the spectra of analyzed species against the reference spectra present in its database. The procedure was carried out following the manufacturer’s guidelines. After diagnosis, the isolated strains from each sample were stored in tubes containing 60% glycerol broth at −20 °C for further availability.

### 2.3. Antimicrobial Susceptibility Testing

For each individual strain, antibiotic susceptibility testing was conducted using the disk diffusion method (Kirby–Bauer) in accordance with EUCAST guidelines [43]. Briefly, a bacterial suspension was prepared in sterile saline (0.9% NaCl, Sigma Aldrich, Darmstadt, Germany) at a 0.5 McFarland density and inoculated onto Mueller–Hinton (MH) agar plates (Merck, Darmstadt, Germany) using the three-section technique. The 14 antimicrobials used were represented by aminoglycosides (gentamicin 10 μg, amikacin 30 μg, tobramycin 10 μg, neomycin 10 μg, kanamycin 30 μg), phenicols (chloramphenicol 30 μg, florfenicol 30 μg), fluoroquinolones (marbofloxacin 5 μg, enrofloxacin 5 μg, ciprofloxacin 5 μg), tetracyclines (tetracycline 30 μg, doxicyline 30 μg), and folate pathway inhibitors (trimethoprim 5 μg, trimethoprim and sulfamethoxazole 1.25/23.75 μg). All commercial antibiotic disks were purchased from Liofilchem, Teramo, Italy, and were radially placed onto the plates.

After sample preparation, incubation was carried out at 35 ± 1 °C for 16–20 h. The inhibition zone diameters for each antibiotic were then measured, and bacterial isolates were classified as susceptible (S), resistant (R), or intermediate (I) according to EUCAST [44] and CLSI VET01S ED7:2024 [45] breakpoints.

### 2.4. Statistical Analysis

Data were collated using Microsoft Office Excel 2021 and statistically analyzed using Epi Info™ 7.2 (CDC, Atlanta, GA, USA) and GraphPad Prism 8 (San Diego, CA, USA). The chi-squared test was employed to evaluate the association between different parameters. Moreover, the Pearson coefficient was also calculated to correlate different variables. *p* < 0.05 was used as the significance threshold for all tests.

## 3. Results

### 3.1. Clinical Presentation

This study included a total of 170 rabbits, which were presented to the clinic with various pathologies. Of the population, a higher proportion of males was observed compared to females, with males constituting 58.24% (*n* = 99) and females 41.76% (*n* = 71). In terms of age distribution, adult rabbits (1–5 years) were the most prevalent group, comprising 42.35% (*n* = 72) of the total population, followed by geriatric rabbits (≥5 years) at 34.71% (*n* = 59) and young rabbits (≤1 year) at 22.94% (*n* = 39). Regarding breed representation, the majority of the rabbits were of the Lionhead breed, accounting for 85.88% (*n* = 146) of the total. Other breeds were less represented, including 3.53% (*n* = 6) Lop, 2.94% (*n* = 5) Dwarf, 2.35% (*n* = 4) Rex, 1.76% (*n* = 3) German Lop, 1.18% (*n* = 2) Mini Rex, and one rabbit (0.59%) from each of the following breeds: American Blue, Angora, Giant White, and Giant Rabbit. In terms of prior antibiotic exposure, 47.06% (*n* = 80) of the rabbits had previously received antimicrobial therapy, while 52.94% (*n* = 90) had not. The pathologies encountered were as follows: dental abscesses were the most common, affecting 35.29% (*n* = 60) of the rabbits. Respiratory affections were the second most prevalent, observed in 28.24% (*n* = 48) of the animals, followed by otitis (13.53%, *n* = 23), ocular signs (9.41%, *n* = 16), skin infections (5.88%, *n* = 10), digestive problems (5.29%, *n* = 9), urinary tract infections (1.76%, *n* = 3), and a single case (0.59%) of an infected surgical pin. Regarding the seasonal distribution of the cases, 34.12% (*n* = 58) of the rabbits were presented during the spring, 17.06% (*n* = 29) in summer, 25.88% (*n* = 44) in autumn, and 22.94% (*n* = 39) in winter. Table 1 summarizes all of the study population’s data.

Out of the 80 cases treated with antibiotics prior to microbiological examination, dental abscesses were the most frequently treated (*n* = 36, 45%), followed by respiratory infections (*n* = 17, 21.25%), otitis (*n* = 10, 12.5%), skin infections (*n* = 7, 8.75%), ocular conditions (*n* = 6, 7.5%), and digestive issues (*n* = 2, 2.5%). Additionally, there was one case of a urinary tract infection (*n* = 1, 1.25%) and one case involving a surgical pin (*n* = 1, 1.25%). The antibiotics used belonged to the following classes: trimethoprim and its derivatives (35.56%), aminoglycosides (18.89%), tetracyclines (14.44%), fluoroquinolones (12.22%), phenicols (6.67%), metronidazole (5.56%), macrolides (3.33%), polymyxins (1.11%), penicillins (1.11%), and cephalosporins (1.11%).

The distribution of pathologies by sex revealed notable differences across various conditions. Of the 60 documented cases of dental abscesses, 46 (76.67%) occurred in males and 14 (23.33%) in females. Respiratory infections were recorded in 48 cases, with 28 (58.33%) affecting males and 20 (41.67%) females. Among the 23 ear-related pathologies, 14 (60.87%) were identified in females and 9 (39.13%) in males. Ocular pathologies were more frequent in females, accounting for 10 out of 16 cases (62.5%), while 6 cases (37.5%) involved males. Similarly, skin infections were more prevalent in females, with 7 of 10 cases (70%) compared to 3 cases (30%) in males. Urinary tract infections were observed in three cases, two (66.67%) in females and one (33.33%) in a male. Digestive pathologies were more common in males, representing six out of nine cases (66.67%), while females accounted for three cases (33.33%). Additionally, a single case of infection associated with a surgical pin was identified in a female. The distribution of pathologies based on age, gender, and season is summarized in Table 2.

The statistical analysis (χ^2^ = 20.3609, df = 7, *p* = 0.0048; Fisher’s Exact Test, *p* = 0.0014) revealed a significant association between sex and the type of pathology in rabbits, with males being more prone to dental (46/99, 46.46%) and respiratory (28/99, 28.28%) conditions, while females showed a higher prevalence of cutaneous (7/71, 9.86%), ocular (10/71, 14.08%), and ear-related (14/71, 19.72%) pathologies. The Chi-Square test (χ^2^ = 23.0547, df = 14, *p* = 0.0594) suggests a moderate association between age and pathology, but it does not reach statistical significance at the 0.05 threshold. Similarly, the test result for season and type of pathology (χ^2^ = 26.2870, df = 21, *p* = 0.1957) indicates no statistically significant association. Although numerical differences are observed, such as a higher frequency of respiratory pathologies in spring, these variations remain statistically insignificant.

### 3.2. Bacterial Identification

Out of the 170 total samples analyzed, 15 (8.82%) were negative, while 155 (91.18%) yielded positive results. The identification of clinical isolates using the MALDI Biotyper^®^ system revealed the presence of bacteria from 23 distinct genera, divided into four categories: Gram-positive cocci (*Aerococcus*, *Enterococcus*, *Glutamicibacter*, *Kocuria*, *Micrococcus*, *Rothia*, *Staphylococcus*, *Streptococcus*), representing 49.5% (*n* = 99) of the isolates; Gram-positive bacilli (*Bacillus*, *Peribacillus*), accounting for 7.5% (*n* = 15); Enterobacteriaceae (*Citrobacter*, *Enterobacter*, *Escherichia*, *Klebsiella*, *Pantoea*, *Proteus*, *Serratia*) with 27.5% (*n* = 55); and non-Enterobacteriaceae (*Acinetobacter*, *Achromobacter*, *Bordetella*, *Moraxella*, *Pasteurella*, *Pseudomonas*) comprising 15.5% (*n* = 31). A total of 200 isolates were obtained, with 114 (57%) being Gram-positive and 86 (43%) Gram-negative, consisting of 45 species. The distribution of each isolated bacterial strain across the various pathologies is detailed in Table 3. Coinfection with two bacterial strains was observed in 27.77% (*n* = 47) of the rabbits, while only 0.65% (*n* = 1) had a coinfection with three distinct bacterial strains. The remaining 71.61% (*n* = 121) exhibited infection with a single bacterial strain.

**Figure 1:**
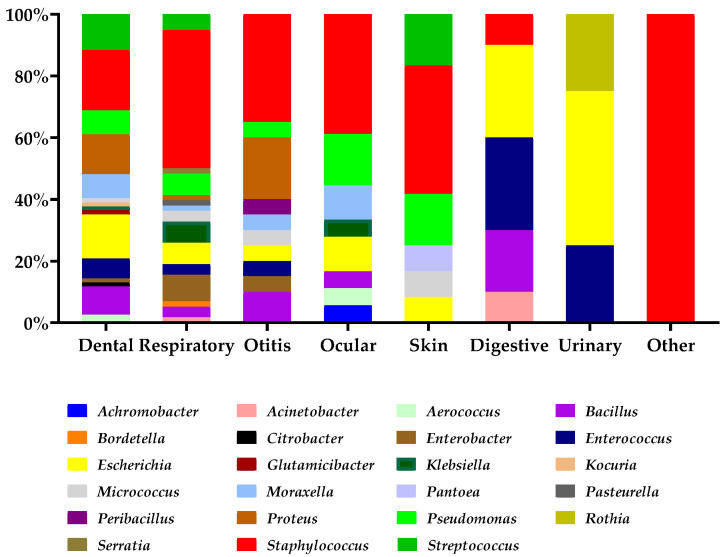
Distribution of bacterial genera based on sample origin in pet rabbits.

The most frequently identified genus was *Staphylococcus* spp., accounting for 62 isolates (31%), followed by *Escherichia* spp. with 24 strains (12%). Among the bacterial species, *Escherichia coli* was the most commonly isolated, representing 24 strains (12%), followed by *Pseudomonas aeruginosa* with 16 isolates (8%). Additionally, *Proteus mirabilis* and *Staphylococcus xylosus* were each detected in 14 cases (7%), while *Staphylococcus sciuri* was identified in 13 samples (6.5%). All bacterial species isolated are presented in Table 3, as well as the distribution of bacterial genera, represented in Figure 1.

### 3.3. Antibiotic Susceptibility Testing

**Figure 2:**
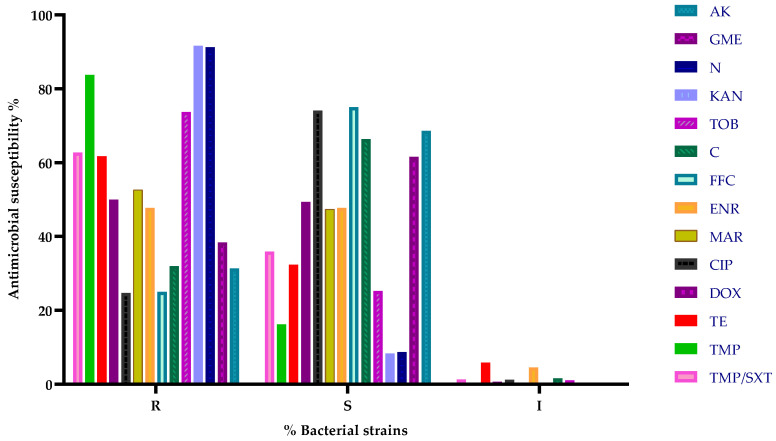
Overall antibiotic susceptibility testing results (% of total bacterial strains). R—resistant; S—susceptible; I—intermediate; AK—amikacin; GME—gentamicin; N—neomycin; KAN—Kanamycin; TOB—tobramycin; C—chloramphenicol; FFC—florfenicol; ENR—enrofloxacin; MAR—marbofloxacin; CIP—ciprofloxacin; DOX—doxycycline; TE—tetracycline; TMP—trimethoprim; TMP/SXT—trimethoprim/sulphamethoxazole.

Antimicrobial susceptibility testing using the disk diffusion method demonstrated high overall efficacy for florfenicol, with 75% of strains exhibiting susceptibility, followed by ciprofloxacin with 74.12% susceptible strains, and amikacin with 68.65% sensitivity. In contrast, significantly higher resistance was observed against other aminoglycosides, such as kanamycin (91.67%) and neomycin (91.30%), as well as trimethoprim (83.78%). A summary of antimicrobial susceptibility results for all tested strains is presented in Figure 2.

**Figure 3:**
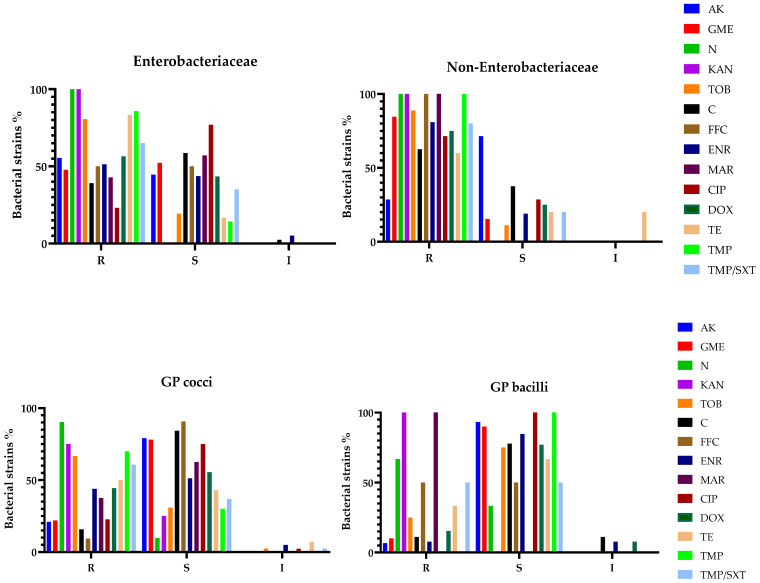
Antibiotic susceptibility testing results for each bacterial group (% of total bacterial strains). R—resistant; S—susceptible; I—intermediate; AK—amikacin; GME—gentamicin; N—neomycin; KAN—Kanamycin; TOB—tobramycin; C—chloramphenicol; FFC—florfenicol; ENR—enrofloxacin; MAR—marbofloxacin; CIP—ciprofloxacin; DOX—doxycycline; TE—tetracycline; TMP—trimethoprim; TMP/SXT—trimethoprim/sulphamethoxazole.

**Figure 4:**
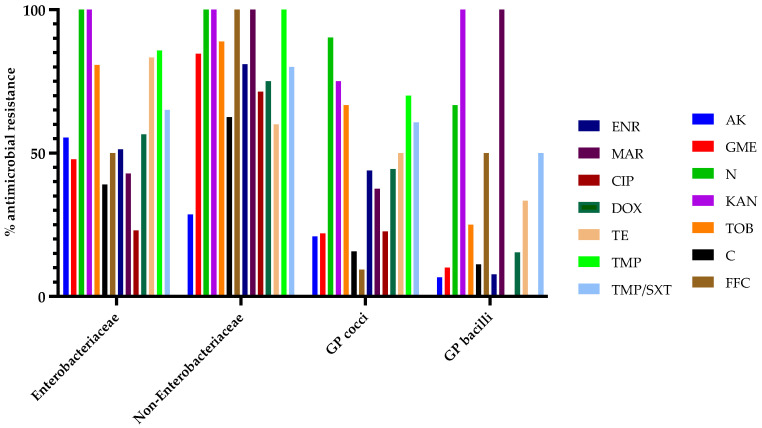
Antimicrobial resistant bacterial strains (%) from each group (Enterobacteriaceae, non-Enterobacteriaceae, GP cocci, GP bacilli). AK—amikacin; GME—gentamicin; N—neomycin; KAN—Kanamycin; TOB—tobramycin; C—chloramphenicol; FFC—florfenicol; ENR—enrofloxacin; MAR—marbofloxacin; CIP—ciprofloxacin; DOX—doxycycline; TE—tetracycline; TMP—trimethoprim; TMP/SXT—trimethoprim/sulphamethoxazole.

The results for antimicrobial susceptibility were also analyzed based on previously established bacterial categories (Enterobacteriaceae, non-Enterobacteriaceae, GP cocci, GP bacilli). Regarding Enterobacteriaceae, bacterial strains within this group displayed total resistance (100%) against neomycin and kanamycin, followed by increased resistance to trimethoprim and tetracycline, with 85.71 and 83.33%, respectively. Bacteria belonging to the non-Enterobacteriaceae group revealed a similar antimicrobial resistance pattern for neomycin and kanamycin, followed by total resistance (100%) for marbofloxacin and trimethoprim. GP cocci showed increased resistance for the following aminoglycosides: neomycin (90.32%), kanamycin (75%), and trimethoprim (70%), as well as tobramycin (66.67%). However, GP bacilli tested as resistant against kanamycin and marbofloxacin (100%), followed by neomycin with 66.67% of strains exhibiting antimicrobial resistance. For a better visualization and data interpretation, the results are graphically presented in Figure 3 and Figure 4.

Moreover, in addition to antimicrobial susceptibility, the presence of multidrug-resistant (MDR) bacteria was also assessed. Isolated strains were classified as MDR whenever tested resistant (non-susceptible) to at least one agent in three or more antimicrobial categories, as proposed in [46]. According to this criterium, 48.50% (*n* = 97) of bacterial strains were categorized as MDR, whereas 51.50% (*n* = 103) were classified as non-MDR, thus testing resistant to one or two different antimicrobial classes. Within the MDR group, 30.93% (*n* = 30) of isolates showed resistance to three antimicrobial categories and 36.08% (*n* = 35) to four antimicrobial categories, whereas 32.99% (*n* = 32) of MDR microorganisms tested non-susceptible to five antimicrobial classes.

**Figure 5:**
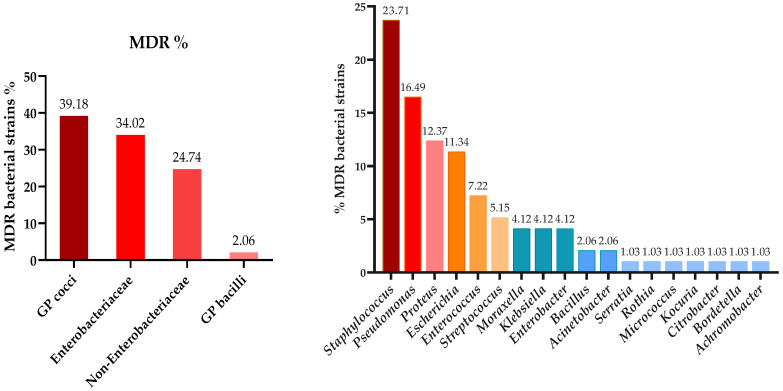
Distribution (%) of MDR isolates on bacterial groups and genera.

The MDR pattern varied across the four bacterial groups, with Gram-positive cocci being the most prevalent multi-resistant strains, closely followed by members of the Enterobacteriaceae group and non-Enterobacteriaceae. Isolates from the following 18 genera were classified as MDR: *Staphylococcus*, *Streptococcus*, *Micrococcus*, *Rothia*, *Enterococcus*, *Kocuria*, *Bacillus*, *Escherichia*, *Enterobacter*, *Klebsiella*, *Proteus*, *Citrobacter*, *Serratia*, *Pseudomonas*, *Achromobacter*, *Bordetella*, *Acinetobacter*, and *Moraxella*. The distribution of MDR strains based on groups and genera is presented in Figure 5. Further details regarding the antibiotic susceptibility testing results are presented in the supplementary material.

## 4. Discussion

The present study provides a detailed overview of the bacteriological diversity in pet rabbits displaying various pathologies, while also examining antibiotic resistance patterns against commonly used antimicrobial agents in veterinary medicine and evaluating the potential zoonotic risk associated with these bacterial strains, emphasizing their implications for both animal and human health.

The scientific literature focused on pathologies and pathogens in pet rabbits remains limited, with relatively few studies published on this subject globally [2,47,48]. In contrast, research investigating various aspects of farm rabbits [39,49,50] and wild populations [51,52,53,54] appears more extensive. This discrepancy highlights the need for further scientific studies on pet rabbits, particularly given their close contact and interaction with humans.

In this research, more males (58.24%) than females (41.76) were presented for consultation, which may have influenced the overall distribution of cases. This trend has also been observed in other studies, where male rabbits were more frequently presented to veterinary clinics, possibly due to behavioral differences, increased territorial aggression, or owner perception of health issues [11,47].

The identification of 23 bacterial genera, with a predominance of Gram-positive cocci (49.5%) and Enterobacteriaceae (27.5%), is consistent with previous studies on bacterial infections in rabbits. *Staphylococcus* spp. was the most frequently isolated genus, accounting for 31% of all isolates, with *Escherichia coli* emerging as the most common species (12%). These findings align with the existing literature, where *Staphylococcus* spp. is often involved in skin infections, abscesses, and otitis in rabbits [3,15,27,55], while *Escherichia coli* is commonly associated with gastrointestinal and urinary tract infections [50,56]. Sex-related differences in bacterial diversity were notable, highlighting potential biological and behavioral factors influencing infection patterns in rabbits. Males were more frequently affected by dental abscesses (76.67%) and respiratory infections (58.33%), which may be linked to anatomical or physiological predispositions, such as differences in skull morphology or dental wear patterns [31]. On the other hand, females showed a higher incidence of ocular disease (62.5%) [57], suggesting possible differences in immune response, grooming habits, or environmental exposure. Even though ear infections had a higher prevalence for female rabbits (60.87%), sex predisposition related to this pathology had not been reported in rabbits [15]. Furthermore, urinary tract infections (66.67%) were also more common in females, which might be associated with anatomical differences in the auditory canal and urinary tract, predisposing them to recurrent infections [58].

The high prevalence of *Staphylococcus xylosus* (7%) and *Staphylococcus sciuri* (6.5%) suggests their potential role as opportunistic pathogens in rabbits, being previously reported in abscesses and skin infections in this species [2,59]. The identification of *Pseudomonas aeruginosa* (8%) and *Proteus mirabilis* (7%) isolates underscores the clinical importance of these bacteria, as both species are known for their intrinsic resistance to multiple classes of antibiotics and their association with chronic infections [60,61]. In fact, several studies associate these bacteria with dental abscesses and respiratory pathologies [2,9,11].

The presence of zoonotic bacteria among the isolates identified in this study highlights the potential risk of bacterial transmission from rabbits to humans, reinforcing the importance of a One Health approach in veterinary and public health. Several species, including *Bordetella bronchiseptica* [62], *Escherichia coli* [39], *Klebsiella pneumoniae* [41], *Pseudomonas aeruginosa* [63], and *Staphylococcus aureus* [55], have been well documented as opportunistic pathogens capable of causing infections in individuals at higher risk. This category includes children under 5, adults over 65, pregnant females, and immunocompromised individuals with HIV/AIDS, cancer, or diabetes or on immunosuppressive medications such as steroids, chemotherapy, and autoimmune disease treatments [40]. Transmission can occur through bites, scratches, the handling of infected animals, or exposure to contaminated bedding and feces [40]. Notably, multidrug-resistant strains, such as *Enterococcus faecium* [50] and *Proteus mirabilis* [60] present a significant challenge for antimicrobial therapy, emphasizing the need for prudent antibiotic use in both human and veterinary medicine.

Regarding the antimicrobial susceptibility testing of bacterial strains isolated from pet rabbit pathologies, the present study revealed a high number of antibiotic-resistant organisms. However, resistance and susceptibility patterns varied across genera and bacterial groups, as previously presented.

As such, the disk diffusion method demonstrated that florfenicol exhibited the highest overall antimicrobial efficacy, with 75% of strains showing susceptibility, followed closely by ciprofloxacin with 74.12%. These results highlight the effectiveness of these antibiotics in combating a broad range of bacterial strains. The high susceptibility to florfenicol could indicate its potential as a treatment option, particularly for infections resistant to other classes of antimicrobials.

On the other hand, a resistance pattern for aminoglycosides was seen, with high resistance observed against several molecules, including kanamycin (91.67%) and neomycin (91.30%), suggesting that these antibiotics may be less effective in treating rabbit bacterial infections. This observation is also confirmed by similar resistance patterns against neomycin and kanamycin seen for Enterobacteriaceae, non-Enterobacteriaceae, and Gram-positive microorganisms as well, with an increased resistance rate compared to in other studies [36]. The high levels of resistance could be linked to the overuse or misuse of aminoglycosides, highlighting the need for alternative therapeutic strategies to prevent further resistance development. Moreover, resistance to trimethoprim and the trimethoprim/sulfamethoxazole combination was notably high overall (83.78% and 62.75%, respectively). Additionally, higher resistance rates were observed across various bacterial groups compared to those reported in other studies [36,64]. These results suggest that previously mentioned antimicrobials may no longer be a reliable treatment for infections caused by the strains tested in this study. Nevertheless, an increased resistance of Enterobacteriaceae to tetracycline was observed (83.33%), consistent with other studies [36].

In the present paper, an interesting antimicrobial susceptibility pattern was observed for tested fluoroquinolones, with an overall increased efficacy of human-use drug ciprofloxacin (74.12%), whereas veterinary-labeled molecules, such as enrofloxacin and marbofloxacin revealed similar results, with 47.74% and 47.37% of total susceptible bacterial strains, indicating a concerning trend in the development of resistance to fluoroquinolones. These findings suggest that antimicrobials within this category (of veterinary use) are still administered as first-line empirical treatment for different rabbit pathologies before requesting a microbiological examination and subsequent antibiotic susceptibility testing. Resistance against fluoroquinolones was previously reported from exotic animals’ isolates, including rabbits [36,64].

This aligns with global trends of increasing resistance to common antimicrobial agents and highlights the importance of continuous surveillance to guide empirical therapy choices, but also the need to explore alternative antibiotics or novel therapeutic approaches. Given the increasing role of companion animals in human households, veterinarians should educate owners on proper hygiene practices to mitigate zoonotic transmission risks, while emphasizing the necessity of continued surveillance, responsible antibiotic use, and improved antibiotic stewardship to prevent the emergence and spread of resistant zoonotic pathogens, especially considering the high levels of resistance observed against key antibiotics like aminoglycosides, trimethoprim, and fluoroquinolones. The findings from this study show a high level of antimicrobial resistance, particularly regarding aminoglycosides and trimethoprim.

Regarding multi-resistance, the findings indicate that nearly half (48.50%) of the bacterial strains isolated in this study were classified as MDR, highlighting a significant challenge in antimicrobial therapy and the importance of addressing resistant bacteria, as they may represent a major public health concern. This finding is consistent with other research data; the authors of [47] reported a similar percentage of MDR strains (49.4%) in their research conducted on pet rabbits. The prevalence of MDR strains reflects an urgent need for alternative treatment options and more effective antimicrobial management. This level of resistance complicates treatment regimens, as it limits the options available for effective therapy. Gram-positive cocci were found to be the most prevalent group of MDR bacteria, which is particularly concerning given the critical role these pathogens play in human infections, such as those caused by *Staphylococcus* and *Enterococcus* [65]. The high prevalence of MDR strains within this group emphasizes the need for targeted surveillance and the development of effective strategies to combat Gram-positive infections.

Bacterial strains from the Enterobacteriaceae and non-Enterobacteriaceae groups also displayed significant MDR patterns. These groups included well-known pathogens such as *Escherichia coli*, *Klebsiella*, and *Pseudomonas aeruginosa*, which are notorious for acquiring resistance to multiple classes of antibiotics, including third-generation cephalosporins, and aminoglycosides [66,67,68]. The emergence of MDR in these groups could lead to limited treatment options, emphasizing the need for continuous surveillance to monitor resistance patterns within these high-risk bacterial groups.

A wide range of microorganisms were identified as MDR in this study, including 18 different bacterial genera, spanning both Gram-positive and Gram-negative species. Notable genera such as *Staphylococcus*, *Enterococcus*, *Klebsiella*, and *Acinetobacter* are recognized for their ability to acquire resistance through various mechanisms, including horizontal gene transfer [69]. This extensive diversity in the genera exhibiting MDR highlights the adaptability of bacteria in response to selective pressure from widespread antimicrobial use. It also raises concerns about the potential for cross-resistance and the horizontal spread of resistance determinants across different bacterial species.

The results of this study further suggest that reliance on existing antibiotics may no longer be sustainable in the long term, as resistance continues to rise. Collaboration between researchers, healthcare providers, and policymakers is essential to accelerate the development of these new treatments. Our findings show that pet rabbits are reservoirs for MDR isolates, particularly staphylococci (*S. sciuri*, *S. xylosus*) and *Pseudomonas aeruginosa*. The present study emphasizes the need for ongoing surveillance and research into the mechanisms of multidrug resistance, especially in key bacterial groups. Future research is more than welcome and should focus on the genetic and environmental factors driving resistance and explore synergistic drug combinations to improve treatment. Large-scale epidemiological studies are also needed to assess the spread of MDR strains and predict future resistance trends.

To the best of our knowledge, this is the first study to isolate and identify *Glutamicibacter protophormiae* as a bacterial etiology in dental abscesses in rabbits. Additionally, this study represents the first investigation in Romania focused on the bacteriological diversity in pet rabbits presenting with various pathologies, offering valuable insights into the spectrum of bacterial species involved, their antimicrobial resistance patterns, and their potential zoonotic risks.

This study has several limitations that should be acknowledged. Firstly, the number of cases in the present research may limit the external validity of our findings and reduce the statistical power of our analyses. Secondly, this study was conducted within a limited geographical area, which may restrict the applicability of the results to broader populations with different epidemiological or clinical characteristics. Finally, only aerobic microorganisms were identified from clinical samples, potentially underrepresenting the role of anaerobic pathogens, thus future research is essential to validate our results.

## 5. Conclusions

This study provides important information about common health issues in rabbits. Dental abscesses were most common in males, while females had more ear, eye, and skin problems. Bacterial infections, mainly from *Staphylococcus* and *Escherichia coli*, were common. No strong links were found between age or season and health problems, but gender differences were clear. The findings show the need for specialized care based on sex and health history, also pointing out the concern of antibiotic resistance, as many bacteria showed resistance to multiple drugs. A novel aspect of this research is that it presents the first identification of *Glutamicibacter protophormiae* as being responsible for a dental abscess in rabbits and is the first study in Romania to explore bacteriological diversity in pet rabbits, highlighting antimicrobial resistance and zoonotic risks.

## Figures and Tables

**Table 1:** Data concerning the population studied based on gender, age, season, pathology, and previous antibiotic exposure.

Rabbits %									Total
Gender	Male 58.24 (*n* = 99)				Female 41.76 (*n* = 71)				170
Age	Young 22.94 (*n* = 39)		Adult 42.35 (*n* = 72)			Geriatric 34.71 (59)			170
Season	Spring 34.12 (*n* = 58)		Summer 17.06 (*n* = 29)		Autumn 25.88 (*n* = 44)		Winter 22.94 (*n* = 39)		170
Pathology	Dental 35.29 (*n* = 60)	Respiratory 28.24 (*n* = 48)	Otitis 13.53 (*n* = 23)	Ocular 9.41 (*n* = 16)	Skin 5.88 (*n* = 10)	Digestive 5.29 (*n* = 9)	Urinary 1.76 (*n* = 3)	Other 0.59 (*n* = 1)	170
Antibioticusage	Yes 47.06 (*n* = 80)				No 52.94 (*n* = 90)				170

**Table 2:** Distribution of pet rabbits’ pathologies according to age, gender, and seasonal variations.

		Dental (*n*)	Respiratory (*n*)	Otitis (*n*)	Ocular (*n*)	Skin (*n*)	Digestive (*n*)	Urinary (*n*)	Other (*n*)	Total
Gender	Male	46 * 46.46% ** 76.67% ***	28 * 28.28% ** 58.33% ***	9 * 9.09% ** 39.13% ***	6 * 6.06% ** 37.50% ***	3 * 3.03% ** 30.00% ***	6 * 6.06% ** 66.67% ***	1 * 1.01% ** 33.33% ***	0 * 0.00% ** 0.00% ***	99 * 100.00% ** 58.24% ***
	Female	14 * 19.72% ** 23.33% ***	20 * 28.17% ** 41.67% ***	14 * 19.72% ** 60.87% ***	10 * 14.08% ** 62.50% ***	7 * 9.86% ** 70.00% ***	3 * 4.23% ** 33.33% ***	2 * 2.82% ** 66.67% ***	1 * 1.41% ** 100.0% ***	71 * 100.00% ** 41.76% ***
	Total	60 * 35.29% ** 100.00% ***	48 * 28.24% ** 100.00% ***	23 * 13.53% ** 100.00% ***	16 * 9.41% ** 100.00% ***	10 * 5.88% ** 100.00% ***	9 * 5.29% ** 100.00% ***	3 * 1.76% ** 100.00% ***	1 * 0.59% ** 100.00% ***	170 * 100.00% ** 100.00% ***
Age	Young (≤1 year)	9 * 23.08% ** 15.00% ***	19 * 48.72% ** 39.58% ***	1 * 2.56% ** 4.35% ***	4 * 10.26% ** 25.00% ***	3 * 7.69% ** 30.00% ***	1 * 2.56% ** 11.11% ***	1 * 2.56% ** 33.33% ***	1 * 2.56% ** 100.0% ***	39 * 100.0% ** 22.94% ***
	Adult (1–5 years)	25 * 34.72% ** 41.67% ***	15 * 20.83% ** 31.25% ***	12 * 16.67% ** 52.17% ***	9 * 12.50% ** 56.25% ***	4 * 5.56% ** 10.00% ***	6 * 8.33% ** 66.67% ***	1 * 1.39% ** 33.33% ***	0 * 0.00% ** 0.00% ***	72 * 100.0% ** 42.35% ***
	Geriatric (≥5 years)	26 * 44.07% 43.33% ***	14 * 23.73% 29.17% ***	10 * 16.95% 43.48% ***	3 * 5.08% 18.75% ***	3 * 5.08% 30.00% ***	2 * 3.39% 22.22% ***	1 * 1.69% 33.33% ***	0 * 0.00% 0.00% ***	59 * 100.0% 34.71% ***
	Total	60 * 35.29% ** 100.0% ***	48 * 28.24% ** 100.0% ***	23 * 13.53% ** 100.0% ***	16 * 9.41% ** 100.0% ***	10 * 5.88% ** 100.0% ***	9 * 5.29% ** 100.0% ***	3 * 1.76% ** 100.0% ***	1 * 0.59% ** 100.0% ***	170 * 100.0% ** 100.0% ***
Season	Spring	25 * 43.10% ** 41.67% ***	17 * 29.31% ** 35.42% ***	9 * 15.52% ** 39.13% ***	3 * 5.17% ** 18.75% ***	2 * 3.45% ** 20.00% ***	1 * 1.72% ** 11.11% ***	1 * 1.72% ** 33.33% ***	0 * 0.00% ** 0.00% ***	58 * 100.0% ** 34.12% ***
	Summer	9 * 31.03% ** 15.00% ***	7 * 24.14% ** 14.58% ***	4 * 13.79% ** 17.39% ***	3 * 10.34% ** 18.75% ***	0 * 0.00% ** 0.00% ***	4 * 13.79% ** 44.44% ***	1 * 3.45% ** 33.33% ***	1 * 3.45% ** 100.0% ***	29 * 100.0% ** 17.06% ***
	Autumn	10 * 22.73% ** 16.67% ***	15 * 34.09% ** 31.25% ***	4 * 9.09% ** 17.39% ***	5 * 11.36% ** 31.25% ***	6 * 13.64% ** 60.00% ***	3 * 6.82% ** 33.33% ***	1 * 2.27% ** 33.33% ***	0 * 0.00% ** 0.00% ***	44 * 100.0% ** 25.88% ***
	Winter	16 * 41.03% ** 26.67% ***	9 * 23.08% ** 18.75% ***	6 * 15.38% ** 26.09% ***	5 * 12.82% ** 31.25% ***	2 * 5.13% ** 20.00% ***	1 * 2.56% ** 11.11% ***	0 * 0.00% ** 0.00% ***	0 * 0.00% ** 0.00% ***	39 * 100.0% ** 22.94% ***
	Total	60 * 35.29% ** 100.0% ***	48 * 28.24% ** 100.0% ***	23 * 13.53% ** 100.0% ***	16 * 9.41% ** 100.0% ***	10 * 5.88% ** 100.0% ***	9 * 5.29% ** 100.0% ***	3 * 1.76% ** 100.0% ***	1 * 0.59% ** 100.0% ***	170 * 100.0% ** 100.0% ***

* number of cases for each pathology; ** percentage of pathology from males/females/young/adults/geriatric/spring/summer/autumn/winter; *** pathology percentage from total cases for gender/age/season.

**Table 3:** Bacterial strains isolated from pet rabbits distributed across different pathologies.

Isolate	%	Dental (*n*)	Respiratory (*n*)	Otitis (*n*)	Ocular (*n*)	Skin (*n*)	Digestive (*n*)	Urinary (*n*)	Other (*n*)
*Achromobacter denitrificans*	0.5 (*n* = 1)	-	-	-	1	-	-	-	-
*Acinetobacter guillouiae*	0.5 (*n* = 1)	-	1	-	-	-	-	-	-
*Acinetobacter johnsonii*	0.5 (*n* = 1)	-	-	-	-	-	1	-	-
*Aerococcus urinae*	0.5 (*n* = 1)	1	-	-	-	-	-	-	-
*Aerococcus viridans*	1 (*n* = 2)	1	-	-	1	-	-	-	-
*Bacillus licheniformis*	3 (*n* = 6)	3	1	1	1	-	-	-	-
*Bacillus pumilus*	4 (*n* = 8)	4	1	1	-	-	2	-	-
*Bordetella bronchiseptica*	0.5 (*n* = 1)	-	1	-	-	-	-	-	-
*Citrobacter braakii*	0.5 (*n* = 1)	1	-	-	-	-	-	-	-
*Enterobacter asburiae*	0.5 (*n* = 1)	-	1	-	-	-	-	-	-
*Enterobacter cloacae complex*	1.5 (*n* = 3)	-	2	1	-	-	-	-	-
*Enterobacter hormaechei*	1 (*n* = 2)	1	1	-	-	-	-	-	-
*Enterobacter kobei*	0.5 (*n* = 1)	-	1	-	-	-	-	-	-
*Enterococcus casseliflavus*	0.5 (*n* = 1)	-	-	-	-	-	1	-	-
*Enterococcus faecalis*	3 (*n* = 6)	4	1	-	-	-	1	-	-
*Enterococcus faecium*	2.5 (*n* = 5)	1	1	1	-	-	1	1	-
*Escherichia coli*	12 (*n* = 24)	11	4	1	2	1	3	2	-
*Glutamicibacter protophormiae*	0.5 (*n* = 1)	1	-	-	-	-	-	-	-
*Klebsiella oxytoca*	2.5 (*n* = 5)	1	3	-	1	-	-	-	-
*Klebsiella pneumoniae*	0.5 (*n* = 1)	-	1	-	-	-	-	-	-
*Kocuria atrinae*	0.5 (*n* = 1)	1	-	-	-	-	-	-	-
*Micrococcus luteus*	2.5 (*n* = 5)	1	2	1	-	1	-	-	-
*Moraxella osloensis*	5 (*n* = 10)	6	1	1	2	-	-	-	-
*Pantoea agglomerans*	0.5 (*n* = 1)	-	-	-	-	1	-	-	-
*Pasteurella canis*	0.5 (*n* = 1)	-	1	-	-	-	-	-	-
*Peribacillus simplex*	0.5 (*n* = 1)	-	-	1	-	-	-	-	-
*Proteus mirabilis*	7 (*n* = 14)	10	1	3	-	-	-	-	-
*Proteus vulgaris*	0.5 (*n* = 1)	-	-	1	-	-	-	-	-
*Pseudomonas aeruginosa*	8 (*n* = 16)	6	4	1	3	2	-	-	-
*Rothia kristinae*	0.5 (*n* = 1)	-	-	-	-	-	-	1	-
*Serratia marcescens*	0.5 (*n* = 1)	-	1	-	-	-	-	-	-
*Staphylococcus aureus*	1 (*n* = 2)	-	2	-	-	-	-	-	-
*Staphylococcus cohnii* ssp. *urealyticus*	0.5 (*n* = 1)	-	-	-	1	-	-	-	-
*Staphylococcus epidermidis*	5 (*n* = 10)	4	-	1	1	3	1	-	-
*Staphylococcus haemolyticus*	1 (*n* = 2)	1	-	-	1	-	-	-	-
*Staphylococcus hominis*	0.5 (*n* = 1)	-	-	-	-	1	-	-	-
*Staphylococcus hominis* ssp. *hominis*	1 (*n* = 2)	1	1	-	-	-	-	-	-
*Staphylococcus saprophyticus*	0.5 (*n* = 1)	-	1	-	-	-	-	-	-
*Staphylococcus sciuri*	6.5 (*n* = 13)	4	7	1	-	-	-	-	1
*Staphylococcus simulans*	3.5 (*n* = 7)	2	3	2	-	-	-	-	-
*Staphylococcus warneri*	4.5 (*n* = 9)	1	4	2	1	1	-	-	-
*Staphylococcus xylosus*	7 (*n* = 14)	2	8	1	3	-	-	-	-
*Streptococcus mitis*	5 (*n* = 10)	9	-	-	-	1	-	-	-
*Streptococcus pneumoniae*	1.5 (*n* = 3)	-	3	-	-	-	-	-	-
*Streptococcus pyogenes*	0.5 (*n* = 1)	-	-	-	-	1	-	-	-
TOTAL	100 (*n* = 200)	38.5 (*n* = 77)	29 (*n* = 58)	10 (*n* = 20)	9 (*n* = 18)	6 (*n* = 12)	5 (*n* = 10)	2 (*n* = 4)	0.5 (*n* = 1)

## Data Availability

The original contributions presented in the study are included in the article, and further inquiries can be directed to the corresponding author.

## Supplementary Materials

The following supporting information can be downloaded at: https://www.mdpi.com/article/10.3390/microorganisms13030653/s1, Table S1: Antimicrobial susceptibility testing results of bacterial strains isolated from pet rabbits.

## Abbreviations

The following abbreviations are used in this manuscript:

MDR	Multidrug resistance
GP	Gram-positive
