# Supplementary material for: Bacterial Diversity in Pet Rabbits: Implications for Public Health, Zoonotic Risks, and Antimicrobial Resistance
Source: Microorganisms. 2025 Mar 13;13(3):653. doi: 10.3390/microorganisms13030653 (PMC11945246; doi:10.3390/microorganisms13030653)
Supplement: Supplementary file 1 [file microorganisms-13-00653-s001.zip › microorganisms-3530500-supplementary.pdf]

Supplementary Table S1. Antimicrobial susceptibility testing results of bacterial strains isolated from pet rabbits

Antimicrobial class/Bacterial strain	Aminoglycosides	Phenicols	Fluoroquinolones	Tetracyclines	FPI	MDR
Achromobacter denitrificans	R	S	S	R	R	Y
Acinetobacter guillouiae	R	R	S	S	R	Y
Acinetobacter johnsonii	R	R	R	R	R	Y
Aerococcus urinae	S	S	R	S	R	N
Aerococcus viridans	S	S	S	S	R	N
Aerococcus viridans	S	S	S	R	R	N
Bacillus licheniformis	R	R	R	R	R	Y
Bacillus licheniformis	S	S	S	S	S	N
Bacillus licheniformis	S	S	S	S	R	N
Bacillus licheniformis	S	S	S	S	R	N
Bacillus licheniformis	S	S	S	S	R	N
Bacillus licheniformis	S	R	S	S	S	N
Bacillus pumilus	R	S	S	S	S	N
Bacillus pumilus	S	S	S	S	S	N
Bacillus pumilus	S	S	S	S	S	N
Bacillus pumilus	S	S	S	S	R	N
Bacillus pumilus	S	R	S	S	S	N
Bacillus pumilus	S	I	S	I	R	N
Bacillus pumilus	R	R	R	R	R	Y
Bacillus pumilus	R	S	S	R	S	N
Bordetella bronchiseptica	R	S	R	S	R	Y
Citrobacter braakii	R	S	R	R	S	Y
Enterobacter asburiae	R	R	S	R	R	Y

Enterobacter cloacae complex	S	R	S	S	R	N
Enterobacter cloacae complex	S	S	S	S	R	N
Enterobacter cloacae complex	S	S	S	S	S	N
Enterobacter hormaechei	R	R	R	R	R	Y
Enterobacter hormaechei	R	S	R	R	R	Y
Enterobacter kobei	R	R	S	R	R	Y
Enterococcus casseliflavus	N/A	R	R	R	R	Y
Enterococcus faecalis	N/A	S	S	S	S	N
Enterococcus faecalis	N/A	S	S	S	R	N
Enterococcus faecalis	N/A	S	S	R	R	N
Enterococcus faecalis	N/A	R	S	R	R	Y
Enterococcus faecalis	N/A	S	R	S	R	N
Enterococcus faecalis	N/A	S	S	R	S	N
Enterococcus faecium	N/A	R	S	R	S	N
Enterococcus faecium	N/A	R	R	S	S	N
Enterococcus faecium	N/A	S	R	R	R	Y
Enterococcus faecium	N/A	R	R	R	R	Y
Enterococcus faecium	N/A	S	S	S	S	N
Escherichia coli	R	S	S	S	R	N
Escherichia coli	R	R	R	R	R	Y
Escherichia coli	R	R	R	R	R	Y
Escherichia coli	R	S	S	S	R	N
Escherichia coli	R	S	R	S	S	N
Escherichia coli	S	S	S	S	S	N
Escherichia coli	R	S	S	S	S	N
Escherichia coli	S	S	S	S	S	N
Escherichia coli	R	S	S	S	S	N
Escherichia coli	S	S	S	S	S	N
Escherichia coli	R	S	S	S	S	N

Escherichia coli	S	R	R	R	S	Y
Escherichia coli	R	S	S	R	R	Y
Escherichia coli	R	S	S	S	R	N
Escherichia coli	R	S	S	R	R	Y
Escherichia coli	R	S	R	R	R	Y
Escherichia coli	R	R	S	R	R	Y
Escherichia coli	R	S	R	R	R	Y
Escherichia coli	R	S	R	R	R	Y
Escherichia coli	R	S	R	S	R	Y
Escherichia coli	S	S	R	S	S	N
Escherichia coli	R	S	S	S	S	N
Escherichia coli	R	S	S	S	R	N
Escherichia coli	R	S	R	R	R	Y
Glutamicibacter protophormiae	R	S	R	S	S	N
Klebsiella oxytoca	R	S	R	S	S	N
Klebsiella oxytoca	R	S	S	S	S	N
Klebsiella oxytoca	R	S	R	R	R	Y
Klebsiella oxytoca	R	S	R	R	R	Y
Klebsiella oxytoca	R	S	R	S	R	Y
Klebsiella pneumoniae	R	S	R	R	R	Y
Kocuria atrinae	R	S	R	R	R	Y
Micrococcus luteus	R	S	S	S	R	N
Micrococcus luteus	S	S	S	S	S	N
Micrococcus luteus	R	R	R	R	R	Y
Micrococcus luteus	R	S	S	S	I	N
Micrococcus luteus	R	S	R	S	S	N
Moraxella osloensis	R	R	R	R	R	Y
Moraxella osloensis	S	S	S	S	R	N
Moraxella osloensis	R	S	R	S	S	N

Moraxella osloensis	R	R	R	R	R	Y
Moraxella osloensis	S	S	S	S	S	N
Moraxella osloensis	S	S	S	R	S	N
Moraxella osloensis	S	S	R	S	R	N
Moraxella osloensis	R	S	R	R	R	Y
Moraxella osloensis	R	R	R	R	R	Y
Moraxella osloensis	R	S	S	S	R	N
Pantoea agglomerans	S	S	S	S	S	N
Pasteurella canis	R	S	S	S	S	N
Peribacillus simplex	S	S	S	S	S	N
Proteus mirabilis	R	R	R	R	R	Y
Proteus mirabilis	R	S	S	R	S	N
Proteus mirabilis	R	S	S	R	R	Y
Proteus mirabilis	R	R	R	R	R	Y
Proteus mirabilis	R	R	S	R	R	Y
Proteus mirabilis	R	R	R	R	R	Y
Proteus mirabilis	R	R	S	R	S	Y
Proteus mirabilis	R	I	R	R	R	Y
Proteus mirabilis	R	R	R	R	R	Y
Proteus mirabilis	S	R	S	R	R	Y
Proteus mirabilis	S	S	S	R	S	N
Proteus mirabilis	R	R	R	R	R	Y
Proteus mirabilis	R	R	S	S	S	N
Proteus mirabilis	R	R	R	R	R	Y
Proteus vulgaris	R	R	S	R	R	Y
Pseudomonas aeruginosa	R	S	S	R	R	Y
Pseudomonas aeruginosa	R	R	R	S	S	Y
Pseudomonas aeruginosa	R	S	R	R	R	Y
Pseudomonas aeruginosa	R	R	R	R	R	Y

Pseudomonas aeruginosa	R	R	R	R	R	Y
Pseudomonas aeruginosa	R	R	R	R	R	Y
Pseudomonas aeruginosa	R	S	R	R	R	Y
Pseudomonas aeruginosa	R	R	R	R	R	Y
Pseudomonas aeruginosa	R	R	R	R	R	Y
Pseudomonas aeruginosa	R	R	R	R	R	Y
Pseudomonas aeruginosa	R	R	R	R	R	Y
Pseudomonas aeruginosa	R	R	R	R	R	Y
Pseudomonas aeruginosa	R	R	R	R	R	Y
Pseudomonas aeruginosa	R	R	R	R	R	Y
Pseudomonas aeruginosa	R	R	R	R	R	Y
Pseudomonas aeruginosa	R	R	R	R	R	Y
Rothia kristinae	S	R	R	R	S	Y
Serratia marcescens	S	R	R	R	R	Y
Staphylococcus aureus	R	S	I	S	R	N
Staphylococcus aureus	R	S	R	R	R	Y
Staphylococcus cohnii ssp. *urealyticus*	S	S	S	S	R	N
Staphylococcus epidermidis	S	S	S	S	R	N
Staphylococcus epidermidis	R	S	R	R	R	Y
Staphylococcus epidermidis	R	S	R	R	R	Y
Staphylococcus epidermidis	S	S	S	S	R	N
Staphylococcus epidermidis	S	S	S	R	S	N
Staphylococcus epidermidis	R	S	S	S	R	N
Staphylococcus epidermidis	R	S	S	R	R	Y
Staphylococcus epidermidis	R	S	S	R	R	Y
Staphylococcus epidermidis	R	S	S	S	S	N
Staphylococcus epidermidis	S	S	S	S	S	N
Staphylococcus haemolyticus	R	S	S	S	R	N

Staphylococcus haemolyticus	R	S	R	S	R	Y
Staphylococcus hominis	R	S	R	R	R	Y
Staphylococcus hominis ssp. *hominis*	R	S	R	R	R	Y
Staphylococcus hominis ssp. *hominis*	R	R	R	R	R	Y
Staphylococcus saprophyticus	S	S	R	S	R	N
Staphylococcus sciuri	R	S	S	R	S	N
Staphylococcus sciuri	S	S	S	S	R	N
Staphylococcus sciuri	R	R	R	R	R	Y
Staphylococcus sciuri	S	S	S	S	S	N
Staphylococcus sciuri	R	S	R	S	R	Y
Staphylococcus sciuri	S	S	S	S	S	N
Staphylococcus sciuri	R	S	R	S	S	N
Staphylococcus sciuri	R	S	S	S	S	N
Staphylococcus sciuri	S	S	R	R	S	N
Staphylococcus sciuri	R	S	S	S	R	N
Staphylococcus sciuri	R	S	R	R	R	Y
Staphylococcus sciuri	R	S	R	S	R	Y
Staphylococcus sciuri	R	S	R	R	S	Y
Staphylococcus simulans	R	S	S	R	S	N
Staphylococcus simulans	S	S	S	S	S	N
Staphylococcus simulans	S	S	S	S	R	N
Staphylococcus simulans	S	S	S	S	S	N
Staphylococcus simulans	R	S	R	S	S	N
Staphylococcus simulans	S	S	S	S	S	N
Staphylococcus simulans	R	S	R	R	R	Y
Staphylococcus warneri	R	S	S	S	R	N
Staphylococcus warneri	S	S	R	S	R	N

Staphylococcus warneri	S	S	S	S	R	N
Staphylococcus warneri	S	S	S	S	R	N
Staphylococcus warneri	S	S	R	R	S	N
Staphylococcus warneri	R	S	R	R	R	Y
Staphylococcus warneri	R	S	I	S	S	N
Staphylococcus warneri	S	S	S	S	S	N
Staphylococcus warneri	S	S	S	S	S	N
Staphylococcus xylosus	R	S	S	S	R	N
Staphylococcus xylosus	R	S	R	R	R	Y
Staphylococcus xylosus	S	S	R	R	R	Y
Staphylococcus xylosus	S	S	S	S	S	N
Staphylococcus xylosus	R	S	S	S	S	N
Staphylococcus xylosus	S	S	S	S	R	N
Staphylococcus xylosus	R	S	R	R	R	Y
Staphylococcus xylosus	R	S	R	R	R	Y
Staphylococcus xylosus	S	S	R	S	S	N
Staphylococcus xylosus	S	S	S	S	S	N
Staphylococcus xylosus	R	S	R	R	R	Y
Staphylococcus xylosus	S	S	S	S	S	N
Staphylococcus xylosus	R	S	R	S	R	Y
Staphylococcus xylosus	R	S	R	R	S	Y
Streptococcus mitis	S	S	S	S	S	N
Streptococcus mitis	R	S	S	S	S	N
Streptococcus mitis	R	R	R	R	R	Y
Streptococcus mitis	S	S	S	S	S	N
Streptococcus mitis	R	S	S	S	R	N
Streptococcus mitis	R	S	R	S	R	Y
Streptococcus mitis	R	S	S	S	I	N
Streptococcus mitis	S	S	R	S	R	N

Streptococcus mitis	R	S	S	R	R	Y
Streptococcus mitis	R	S	R	R	R	Y
Streptococcus pneumoniae	S	S	R	S	R	N
Streptococcus pneumoniae	S	S	S	S	R	N
Streptococcus pneumoniae	R	S	R	R	S	Y
Streptococcus pyogenes	S	S	S	S	S	N

MDR – multidrug resistant

FPI – folate pathway inhibitors

R – resistant

I - intermediate

S – sensitive

Y – yes

N – no

N/A – not applicable (intrinsic antibiotic resistance)
